# *Photorhabdus viridis* sp. nov. Isolated from *Heterorhabditis zealandica* Entomopathogenic Nematodes

**DOI:** 10.1007/s00284-024-03935-y

**Published:** 2024-10-23

**Authors:** Ricardo A. R. Machado, Antoinette P. Malan, Joaquín Abolafia, Jaspher Ewany, Aashaq Hussain Bhat, S. Patricia Stock

**Affiliations:** 1 Experimental Biology Research Group, Institute of Biology, University of Neuchâtel, Rue Emile-Argand 11, 2000 Neuchâtel, Switzerland; 2 Department of Conservation Ecology and Entomology, Stellenbosch University, Private Bag X1, Stellenbosch, 7602 Matieland South Africa; 3 Departamento de Biología Animal, Biología Vegetal y Ecología, Universidad de Jaén, Campus ‘Las Lagunillas’, Jaén, Spain; 4 Department of Biosciences and University Center for Research and Development, Chandigarh University. Gharuan, Mohali, Punjab 140413 India; 5 School of Animal and Comparative Biomedical Sciences, University of Arizona, Tucson, Arizona USA

## Abstract

A novel bacterial species, *Photorhabdus viridis* sp. nov., represented by strain Green^T^, isolated from *Heterorhabditis zealandica* MJ2C entomopathogenic nematodes, is described. Phylogenetic reconstructions using 16S rRNA gene sequences show that strain Green^T^ is closely related to *P. thracensis* DSM 15199^ T^. The 16rRNA gene sequences of these two strains are 98.8% identical. Phylogenetic reconstructions using whole-genome sequences show that strain Green^T^ is closely related to *P. tasmaniensis* DSM 22387^ T^, *P. thracensis* DSM 15199^ T^, and *P. temperata* DSM 14550^ T^. Digital DNA-DNA hybridization (dDDH) values between strain Green^T^ and its three more close relative species, *P. tasmaniensis* DSM 22387^ T^, *P. thracensis* DSM 15199^ T^, and *P. temperata* DSM 14550^ T^, are 49%, 59%, and 59%, respectively. In addition, average nucleotide identity (ANI) values between Green^T^ and *P. tasmaniensis* DSM 22387^ T^, *P. thracensis* DSM 15199^ T^, and *P. temperata* DSM 14550^ T^ are 92.4%, 94.4%, and 94.6%, respectively. The novel species also differs in their biochemical capacities from the biochemical capacities of their more closely related taxa. The following biochemical tests may be particularly useful in this context: Arginine dihydrolase, gelatinase, and glucose and mannitol oxidation. Given the clear phylogenetic separation, the sequence divergence values, and the phenotypic differences, we conclude that strain Green^T^ represents a novel bacterial species, for which we propose the name *Photorhabdus viridis* sp. nov. with Green^T^ (= CCM 9407^ T^ = CCOS 2117^ T^ = MJ2C^T^) as the type strain. Our study contributes to a better understanding of the taxonomy and biodiversity of an important bacterial group with great biotechnological and agricultural potential.

**Supplementary Information:**

The online version contains supplementary material available at 10.1007/s00284-024-03935-y.

## Introduction

Species of the bacterial genus *Photorhabdus* are symbiotically associated with *Heterorhabditis* entomopathogenic nematodes (EPNs) [1–4]. These organisms are cosmopolitan and have been isolated from different locations around the globe, except Antarctica [5]. The nematodes colonize soil-borne insects and release their symbiotic bacteria, which kill the colonized insect using different toxins and digestive enzymes [6–8]. The nematodes and the bacteria then proliferate in the insect cadavers [9]. Upon resource depletion, the nematodes and the bacteria re-establish symbiosis and abandon the cadaver in search of a new host [10]. These lethal organisms are important biological control agents and are widely used to control agricultural pests [11–14]. In addition, *Photorhabdus* produces different bioactive compounds, including antibiotics, making these organisms of significant biotechnological and medical relevance [15].

The type strain of the type species of the genus *Photorhabdus*, Hb^T^, was isolated from *Heterorhabditis bacteriophora* EPNs collected in Brecon (Australia). This strain was initially classified in the genus *Xenorhabdus*, together with other bacterial symbionts isolated from *Steinernema* EPNs [16, 17]. To harmonize the taxonomy of the bacteria symbiotically associated with both genera of EPNs, Boemare et al. (1993) proposed to accommodate the bacterial symbionts of *Steinernema* nematodes in the bacterial genus *Xenorhabdus*, and to transfer the bacterial species associated with *Heterorhabditis* nematodes to a newly created genus, *Photorhabdus* [18]. Since its creation, several *Photorhabdus* species and subspecies have been described [17–35]*.* Currently, the *Photorhabdus* genus contains 31 taxa. From these, there are 23 child taxa with validly published and correct names, 6 of which are divided into different subspecies, and 1 child taxa, *P. africana*, whose name is in the process of validation. The complete list can be accessed here: https://lpsn.dsmz.de/genus/photorhabdus [36].

The aim of this study was to characterize a novel *Photorhabdus* species isolated from *Heterorhabditis zealandica* Poinar entomopathogenic nematodes. Our study contributes to a better understanding of the taxonomy and biodiversity of this bacterial group, which is of biotechnological and agricultural relevance. This, in turn advances our efforts toward developing more biocontrol tools for sustainable and environmentally friendly agriculture.

## Material and Methods

### Origin and Culturing of Bacterial Strains

**Fig. 1:**
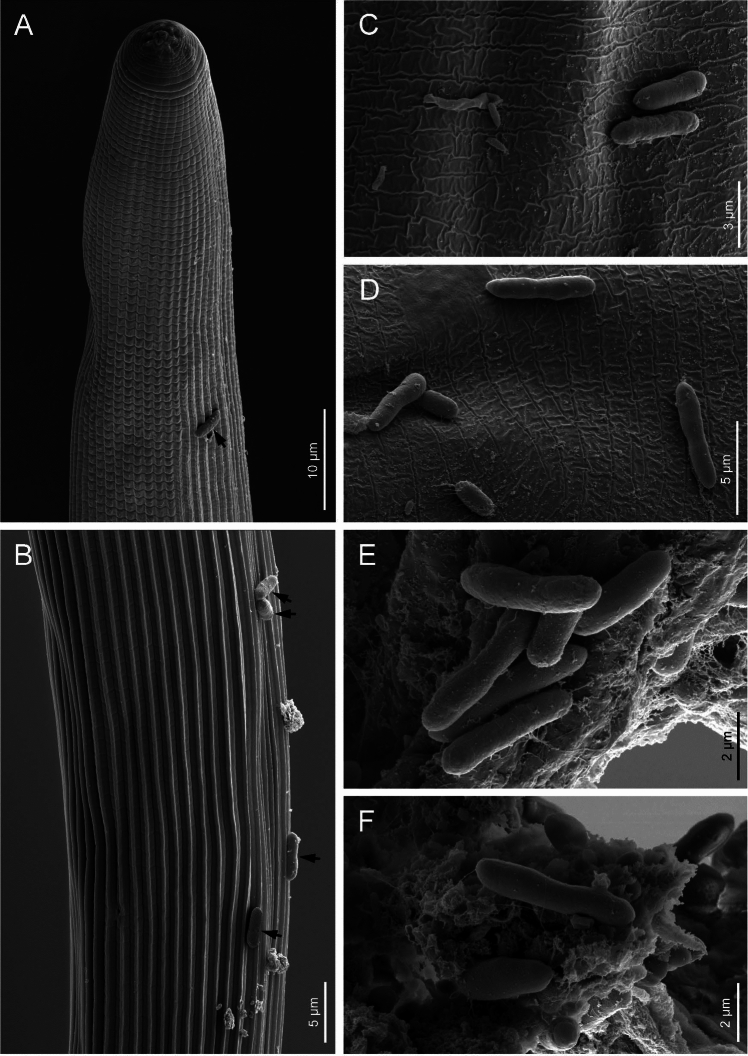
**a** Scanning electron microscopy (SEM) photographs of *Photorhabdus viridis* sp. nov. Green^T^ and *Heterorhabditis zealandica* infective juvenile nematodes. Black arrows point to bacterial cells. **a**, **b** Nematode infective juvenile with bacterial cells on the cuticle. C-F) Closeups of nematode infective juvenile cuticle with bacterial cells attached

**Fig. 2:**
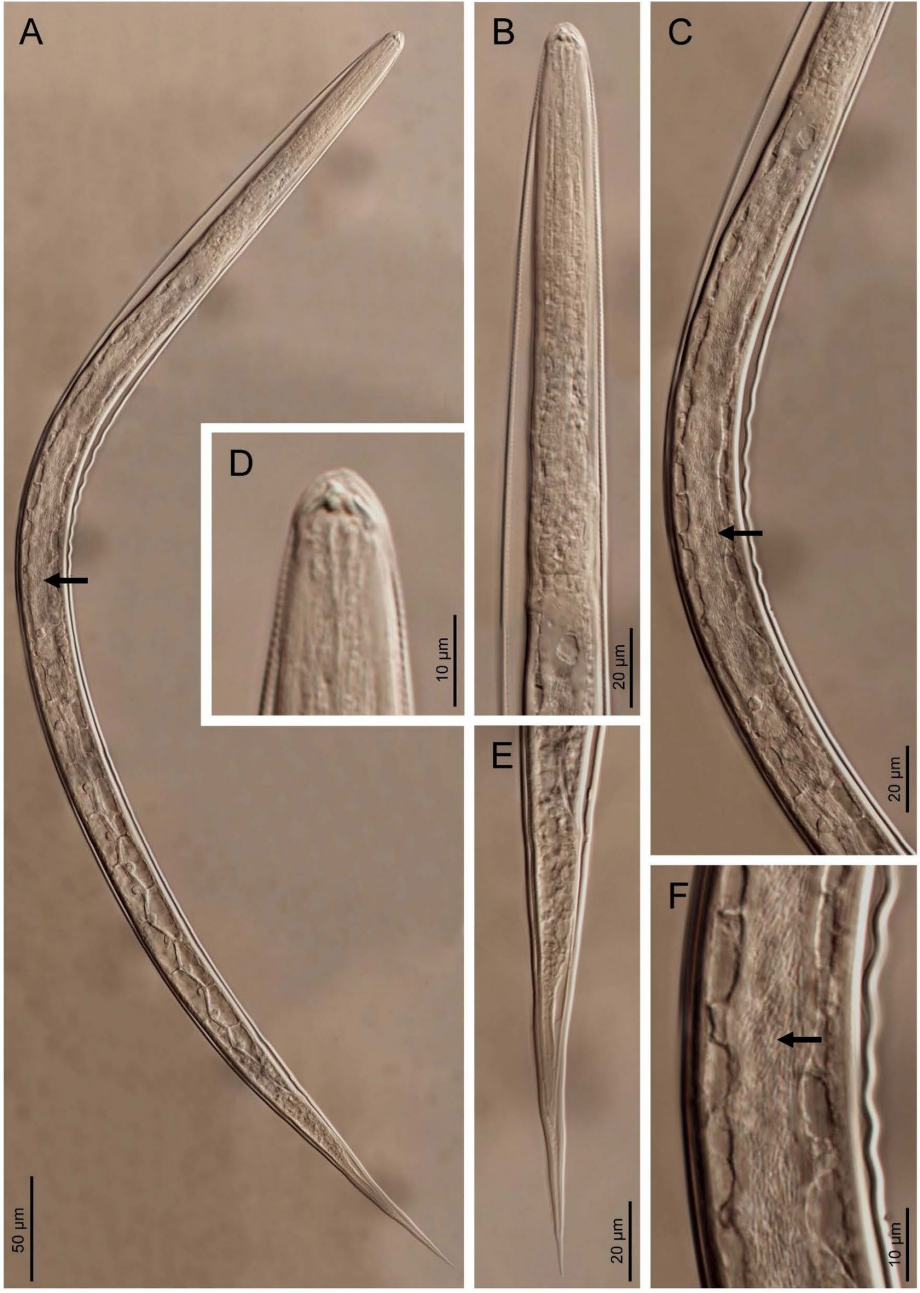
**a–f** Light microscopy (LM) photographs of *Photorhabdus viridis* sp. nov. Green^T^ and *Heterorhabditis zealandica* infective juvenile nematodes. Black arrows point to bacterial cells

The bacterial strain Green^T^ (= MJ2C^T^) was isolated from *Heterorhabditis zealandica* MJ2C nematodes [37](Figs. 1, 2). Using the sequences of housekeeping genes, this bacterial strain was found to be closely related to *P. thracensis* and was therefore regarded as belonging to this species, rather than representing a new species, as shown in this study based on a larger genomic dataset [38]. The bacterial strains were cultured and maintained on lysogeny Broth (LB) agar plates (Sigma-Aldrich, Switzerland) at 28–30 °C.

### Bacteria molecular characterization

To molecularly characterize the strain Green^T^, phylogenetic relationships were reconstructed using 16S rRNA gene and whole-genome sequences. In addition, sequence similarity scores were calculated as described below.

### 16S rRNA Gene Sequencing

The 16S rRNA gene sequences were obtained as previously described [35]. Briefly, genomic DNA was extracted and purified using the GenElute Bacterial Genomic DNA Kit (Sigma–Aldrich, Switzerland) following the manufacturer’s instructions. The 16S rRNA gene was then amplified by polymerase chain reaction (PCR) using the following universal primers: 27F (5′-AGAGTTTGATCMTGGCTCAG-3′) and 1525R (5′-AAGGAGGTGWTCCARCC-3′). The cycling conditions were as follows: 1 cycle at 94 °C for 10 min, followed by 40 cycles at 94 °C for 60 s, 55 °C for 60 s, 72 °C for 60 s, and a final extension at 72 °C for 5 min [39–41]. PCR products were separated by electrophoresis in a 1% TAE-agarose gel stained with GelRed nucleic acid gel stain (Biotium), gel-purified using the QIAquick Gel Purification Kit (Qiagen), and sequenced by Sanger sequencing (Microsynth AG, Balgach, Switzerland). The obtained sequences were manually curated using Bioedit 7.2.5 [42]. In addition, 16S rRNA sequences were obtained directly from the whole-genome sequences using the bacterial ribosomal RNA predictor Barrnap [43]. Phylogenetic relationships were reconstructed using the Maximum Likelihood method based on the Kimura 2-parameter model in MEGA7 as described above [44–46]. The accession numbers of the sequences used for these analyses are shown in Table S1.

### Whole Genome Sequencing

Genome sequences of strain Green^T^ were obtained as previously described [47, 48]. Briefly, gDNA was extracted and purified using the GenElute Bacterial Genomic DNA Kit (Sigma–Aldrich, Switzerland), following the manufacturer’s instructions. The resulting DNA was used for library preparation using the TruSeq DNA PCR–Free LT Library Prep (FC–121–3003) kit. Indexed libraries were pooled at equimolar concentrations and sequenced (2 × 150 bp) on an Illumina HiSeq 3000 instrument. Genomes were assembled using the Bactopia pipeline [49]. To this end, raw Illumina reads were quality trimmed using Trimmomatic 0.39 [50]. The trimmed reads were assembled with SPAdes 3.14.1 using k–mer sizes of 31, 51, 71, 91, and 111 bp [51]. Scaffolds with a mean read–depth smaller than 20% of the median read–depth of the longer scaffolds (≥ 5,000 bp) were filtered out. Minor assembly errors were corrected using Pilon 1.22 [52]. Completeness and contamination of the assembled genomes were assessed using CheckM v1.2.2 with default parameters [53].

### Core Genome-Based Phylogenetic Reconstructions and Sequence Comparisons

To reconstruct whole-genome-based phylogenetic relationships, genomes were first aligned using Roary 3.13.0. Genes to be considered core had to be present in 85% of the genomes with an 85% protein identity. Obtained alignments were used to build phylogenetic trees using FastTree 2.1.10 based on the Generalized Time Reversible Model (GTR). Graphical representation and edition of the phylogenetic trees were performed with Interactive Tree of Life (v3.5.1) [54, 55]. Digital DNA-DNA hybridization (dDDH) values were used to determine pairwise whole-genome sequence similarities. These values were calculated using the GBPD (Genome Blast Distance Phylogeny) method through the Genome-to-Genome Distance Calculator 2.1 and formula 2 of the Deutsche Sammlung von Mikroorganismen und Zellkulturen (DSMZ) web service (http://ggdc.dsmz.de) using default parameters [56–59]. Digital DNA-DNA hybridization (dDDH) values of 70% and 79% delimit species and subspecies boundaries, respectively [26, 56, 60]. Average nucleotide identify (ANI) values were calculated using FastANI [61]. ANI values of 95–96% delimit species boundaries. These overall genomic relatedness indices (OGRI) thresholds are to be used as a reference and not as absolute values [62]. Additional data, such as phylogenomic reconstructions, can justify the separation of species above the recommended thresholds [62]. The accession numbers of the sequences used for these analyses are shown in Table S1.

### Genomic Comparative Analyses

Genomic comparative analyses were conducted to annotate and determine the presence or absence of genes involved in antibiotic resistance or in the production of specialized metabolites Draft genome assemblies were aligned against the Comprehensive Antibiotic Resistance Database (“CARD”) [63–68] and against the Antibiotics and Secondary Metabolite Analysis Shell (antiSMASH) database [69, 70]. Genes that passed the threshold values (antibiotic resistance: ≥ 70% nucleotide identity and ≥ 50% coverage; antiSMASH: ≥ 50% nucleotide identity) were considered present in the genome [69, 70]. Below this threshold, genes were considered absent or non-functional.

### Physiological, Biochemical and Morphological Characterization

The following bacterial species were physiologically, biochemically, and morphologically characterized: *P. viridis* sp. nov. Green^T^, *P. tasmaniensis* DSM 22387^ T^, *P. thracensis* DSM 15199^ T^, and *P. temperata* DSM 14550^ T^*.* All the strains were evaluated in parallel, under identical conditions. In all experiments, bacterial cultures from single primary colonies were used. Primary forms were determined by examining colony consistency (mucoid), bioluminescence, and pigment production [71]. The selected colonies were further sub-cultured and maintained on Lysogeny Broth (LB) agar plates at 28–30 °C. Cell morphology was observed under a Kern transmitted light microscope at 1000 × magnification, with cells grown for two days at 28 °C on LB agar plates. The optimum temperature for bacterial growth was evaluated on LB plates at 18 °C, 23 °C, 28 °C, 32 °C, 37 °C, and 42 °C. Growth in media with varying salt concentrations and pH levels was evaluated in 3 mL of LB medium using 14 mL Falcon tubes. Three NaCl concentrations were used: 1% (Regular LB medium), 2%, and 3% (w/v). Three pH levels were used: 5, 7, and 9. Each tube was inoculated with 0.3 mL (OD_600_ = 1) of an overnight bacterial culture, then incubated for 24 h at 28 ºC and 180 rpm, and finally the OD_600_ was measured using a spectrophotometer. Four tubes per treatment were evaluated. Cytochrome oxidase production was tested on discs containing *N,N*-dimethyl-p-phenylenediamine oxalate and α-naphthol (Sigma-Aldrich, Switzerland). Catalase activity was determined by adding a drop of 10% (v/v) H_2_O_2_ into 50 µL of a 16 h-old liquid LB-bacterial culture. Biochemical characterization was carried out using the API20E system (bioMérieux, Inc. Durham, NC) according to the manufacturer’s instructions. To this end, bacteria were grown for 16 h at 28 °C on LB agar Petri plates. Then, one single colony was re-suspended in 5 mL of 0.85% (w/v) NaCl. The resulting bacterial solution was used to inoculate the different microtubes containing the biochemical tests. Samples were incubated at 28 °C, and results were evaluated after 24 h. Bioluminescence production was evaluated by making photographs of bioluminescence on 3-week-old LB agar-cultured bacteria using an Amersham Imager 680 instrument (Cytiva, US).

### Ecological Characterization

To evaluate the entomopathogenic potential, bacteria were cultured overnight in LB liquid medium. The bacterial cultures were then collected, and their optical densities at 600 nm (OD_600_) were measured. Subsequently, all cultures were diluted to reach an OD_600_ = 1. The resulting cultures were further serially diluted to obtain bacterial solutions with an OD_600_ = 0.01. Ten µL of the resulting bacterial solutions were injected into third-instar *G. mellonella* larvae. Eight larvae per bacterial strain were injected (n = 8). Control insects were injected with pure LB. Mortality was evaluated every 12 h for 3 days.

## Results and Discussion

### 16S rRNA Gene-Based Phylogenetic Reconstruction and Sequence Comparisons

Phylogenetic reconstructions using 16S rRNA gene sequences show that strain Green^T^ is closely related to *P. thracensis* DSM 15199^ T^ (Fig. S1). The 16rRNA gene sequences of strain Green^T^ and *P. thracensis* DSM 15199^ T^ are 98.8% identical (Fig. S2).

### Core genome-Based Phylogenetic Reconstructions and Sequence Comparisons

**Fig. 3:**
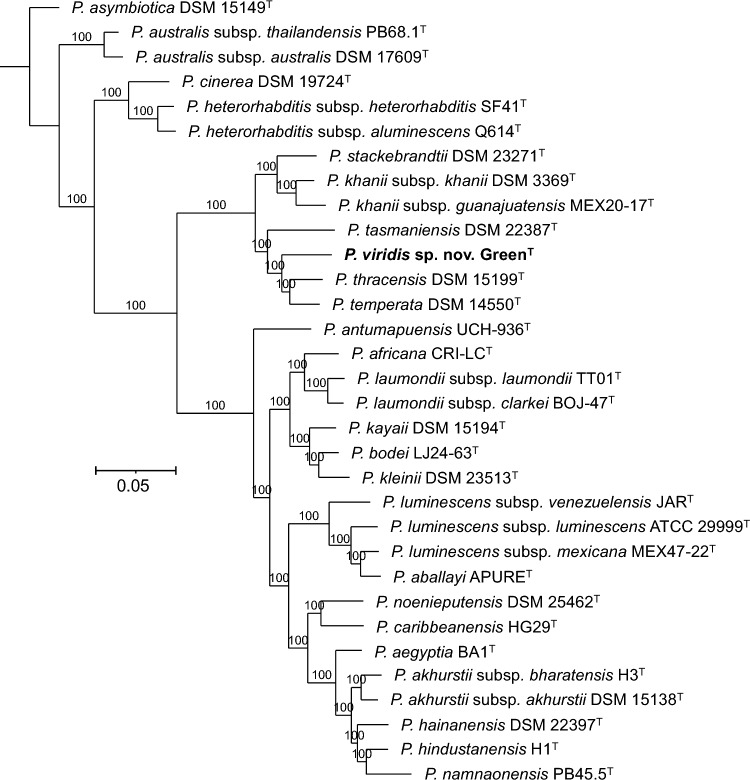
Phylogenetic reconstruction based on core-genome sequences of *Photorhabdus* type strains with validly published names. A total of 4,359,402 nucleotide positions (4388 core genes) were used in the analyses. Numbers at the nodes represent SH-like branch supports. Bar represents 0.05 nucleotide substitutions per sequence position. Accession numbers of the genome sequences used for the reconstruction are shown in Table S1

**Fig. 4:**
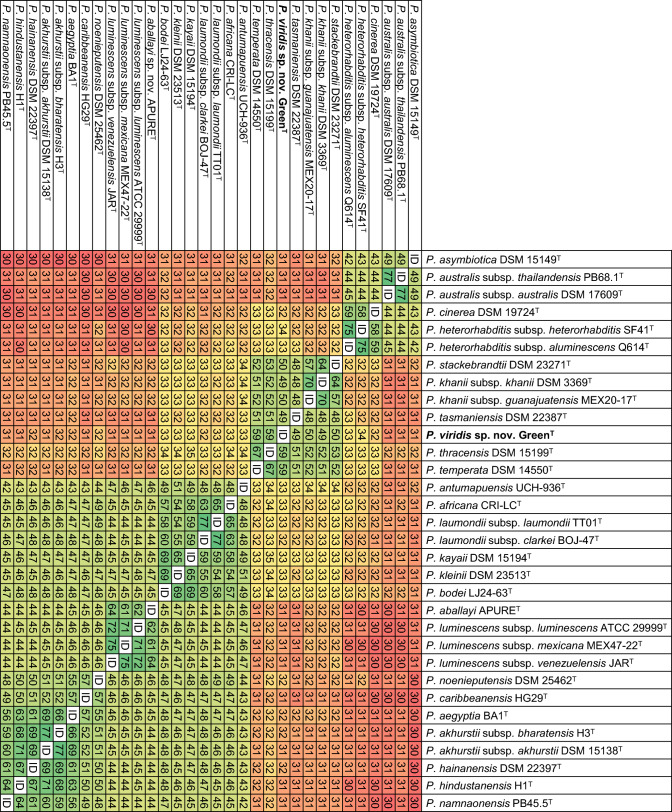
Pairwise comparison of digital DNA-DNA Hybridization (dDDH) values (%) of *Photorhabdus* type strains with validly published names. Accession numbers of gene sequences used are shown in Table S1

In core-genome phylogenies, strain Green^T^ forms a distinct clade together with *P. tasmaniensis* DSM 22387^ T^, *P. thracensis* DSM 15199^ T^, and *P. temperata* DSM 14550^ T^ (Fig. 3). Digital DNA-DNA hybridization (dDDH) values between Green^T^ and *P. tasmaniensis* DSM 22387^ T^, *P. thracensis* DSM 15199^ T^, and *P. temperata* DSM 14550^ T^ are 49%, 59%, and 59%, respectively (Fig. 4). In addition, average nucleotide identity (ANI) values between strain Green^T^ and *P. tasmaniensis* DSM 22387^ T^, *P. thracensis* DSM 15199^ T^, and *P. temperata* DSM 14550^ T^ are 92.4%, 94.4%, and 94.6%, respectively (Figure S3). Given the clear phylogenetic separation and the sequence divergence values, we conclude that strain Green^T^ represents a novel bacterial species, for which we propose the name *Photorhabdus viridis* sp. nov. with Green^T^ (= CCM 9407^ T^ = CCOS 2117^ T^ = MJ2C^T^) as the type strain.

## Genomic Features

### Genome size, Nucleotide Composition, and Number of Predicted Coding Genes

The genome of *P. viridis* sp. nov. Green^T^ is predicted to contain 5115 protein-coding genes, a G + C content of 43.64%, and a total size of 5.12 Mbp (Tables S2 and S3). These values fall within the typical range observed for many of the species of the genus (Table S2). According to CheckM (v1.2.2), the assembled genome of *P. viridis* sp. nov. Green^T^ is 100% complete, and has 0.6% of contamination.

### Predicted Antibiotic Resistance Phenotypes

In silico analyses allow to predict that *P. viridis* sp. nov. Green^T^ and their most closely related species may be resistant to multiple antibiotics, which is a common trait in this bacterial genus (Tables S4). However, *P. viridis* sp. nov. Green^T^ may be susceptible to elfamycins (Table S4).

### Predicted Biosynthetic Capacity

In silico analyses using the antibiotics and secondary metabolite analysis shell (antiSMASH) database uncovers the presence of biosynthetic gene clusters dedicated to the production of several polyketides and non-ribosomal peptides in the genome of *P. viridis* sp. nov. Green^T^ including ambactin, andrimid, bovienimide A, holomycin, kolossin, photobactin, pyrrolizixenamide A, rhizomide A-C, ririwpeptide A-C, and stechlisins. Several of these metabolites are also produced by closely related species (Table S5).

### Physiological and Biochemical Characteristics

Biochemical tests show that *P. viridis* sp. nov. Green^T^ exhibits biochemical capacities that are similar to those of its more closely related species (Table 1). However, *P. viridis* sp. nov. Green^T^ shows also unique biochemical capacities that differ from the biochemical capacities of their most closely related taxa*.* Arginine dihydrolase, gelatinase, and glucose and mannitol oxidation allow to differentiate *P. viridis* sp. nov. Green^T^ from its more closely related taxa (Table 1). Moreover, *P. viridis* sp. nov. Green^T^ produces bioluminescence, which is a typical characteristic of this bacterial genus (Fig. S4). Additional biochemical tests potentially useful to differentiate the different taxa are available in previous literature [25, 30, 33].

**Table 1:** Phenotypic characters of *Photorhabdus viridis* sp. nov. Green^T^, and their more closely related taxa: *P. tasmaniensis* DSM 22387^ T^, *P. thracensis* DSM 15199^ T^, and *P. temperata* DSM 14550^ T^*.* + : positive reaction. −: negative reaction

Biochemical test	Green^T^	DSM 22387^ T^	DSM 15199^ T^	DSM 14550^ T^
β-galactosidase	−	−	−	−
Arginine dihydrolase	+	−	−	−
Lysine decarboxylase	−	−	−	−
Ornithine decarboxylase	−	−	−	−
Citrate utilization	+	−	+	−
H_2_S production	−	−	−	−
Urease	+	−	−	+
Tryptophan deaminase	−	−	−	+
Indole production	+	+	−	−
Acetoin production	−	−	−	−
Gelatinase	−	+	+	+
Glucose oxidation	−	+	+	+
Mannitol oxidation	+	−	−	−
Inositol oxidation	−	−	−	−
Sorbitol oxidation	−	−	−	−
Rhamnose oxidation	−	−	−	−
Sucrose oxidation	−	−	−	−
Melibiose oxidation	−	−	−	−
Amygdalin oxidation	−	−	−	−
Arabinose oxidation	−	−	−	−
(Cytochrome) oxidase	−	−	−	−
Catalase	+	+	+	+
NO_2_ production	−	−	−	−
NO_2_ reduction to N_2_ gas	−	−	−	−

### Ecological Characterization

When injected into the hemocoel of *G. mellonella* larvae, *P. viridis* sp. nov. Green^T^ rapidly killed the infected insects (Figure S5). This strain killed 100% of the infected insects within 48 h (Figure S5).

## Protologue

### Description of *Photorhabdus viridis* sp. nov.

(vi’ri.dis. L. fem. adj. *viridis*, green, because *Galleria mellonella* (Lepidoptera: Phylaridae) insects infected by this bacterial strain or killed by its nematode host turn greenish in color). Cells are rod-shaped of approx. 0.5–0.8 µm wide and 4.5–5.0 µm long. Colonies are mucoid, circular, with slightly irregular margins, and appear greenish-yellow in color, occasionally becoming brownish in cultures older than 7 days, with a diameter of approx. 1–2 mm after 48 h of growth on LB agar. Produce bioluminescence. Bacterial growth in liquid LB medium occurs at temperatures between 18 and 37 °C. Optimal temperature for growth is 28–30 °C. Bacterial growth is strongly impaired at temperatures above 37 °C and does not grow at temperatures above. Bacteria grow in liquid LB with pH between 5 and 9 (optimum 7). Bacterial growth occurs in LB medium containing between 1–2% (w/v) NaCl (optimum 1%). Bacterial growth is inhibited in LB containing > 2% NaCl. Negative for cytochrome oxidase, β-galactosidase, lysine decarboxylase, ornithine decarboxylase, tryptophan deaminase, gelatinase, and hydrogen sulphide and acetoin production. Positive for catalase, arginine dihydrolase, citrate utilization, urease, indole production, and mannitol oxidation. Does not oxidize inositol, glucose, sorbitol, rhamnose, sucrose, melibiose, amygdalin or arabinose. The type strain was isolated from *Heterorhabditis zealandica* nematodes. Whole genome sequences of strain Green^T^ are deposited in the National Center for Biotechnology Information (NCBI) databank under the accession number JBEJZY01, and the 16S rRNA gene sequence under the accession number PP911496. The assembled genome contains 5,122,085 base pairs, 5115 proteins, and a 43.64% G + C content. The type strain of the species, Green^T^ (= MJ2C^T^), was deposited in the Czech Collection of Microorganisms (CCM) and in the national Culture Collection of Switzerland (CCOS) under the following accession numbers: CCM 9407^ T^ and CCOS 2117^ T^, respectively.

## Supplementary Information

Below is the link to the electronic supplementary material.

Supplementary file1 (PDF 677 KB)
