# Supplementary material for: Photorhabdus viridis sp. nov. Isolated from Heterorhabditis zealandica Entomopathogenic Nematodes
Source: Curr Microbiol. 2024 Oct 23;81(12):423. doi: 10.1007/s00284-024-03935-y (PMC11499390; doi:10.1007/s00284-024-03935-y)
Supplement: Supplementary file 1 — Supplementary file1 (PDF 677 KB) [file 284_2024_3935_MOESM1_ESM.pdf]

-SUPPLEMENTARY MATERIAL-

Photorhabdus viridis **sp. nov. isolated from** ***Heterorhabditis zealandica*** **entomopathogenic nematodes**

Ricardo A. R. Machado[1]*, Antoinette P. Malan[2], Joaquín Abolafia[3], Jaspher Ewany[1], Aashaq H. Bhat[4], Patricia Stock[5]

[1]*Experimental Biology Research Group. Institute of Biology, University of Neuchâtel. Neuchâtel, Switzerland.*

[2]*Department of Conservation Ecology and Entomology, Stellenbosch University. Private Bag X1, 7602 Matieland, South Africa.*

[3]*Departamento de Biología Animal, Biología Vegetal y Ecología, Universidad de Jaén, Campus 'Las Lagunillas'. Jaén, Spain.*

[4]*Department of Biosciences and University Center for Research and Development, Chandigarh University. Gharuan, Mohali, Punjab, 140413, India.*

[5]*School of Animal and Comparative Biomedical Sciences, University of Arizona. Tucson, Arizona.*

*Corresponding author: Ricardo A. R. Machado (ricardo.machado@unine.ch). Experimental Biology Research Group. Institute of Biology. University of Neuchâtel. Rue Emile-Argand 11, 2000 Neuchâtel, Switzerland. +41(0)327183076.

-SUPPLEMENTARY FIGURES-

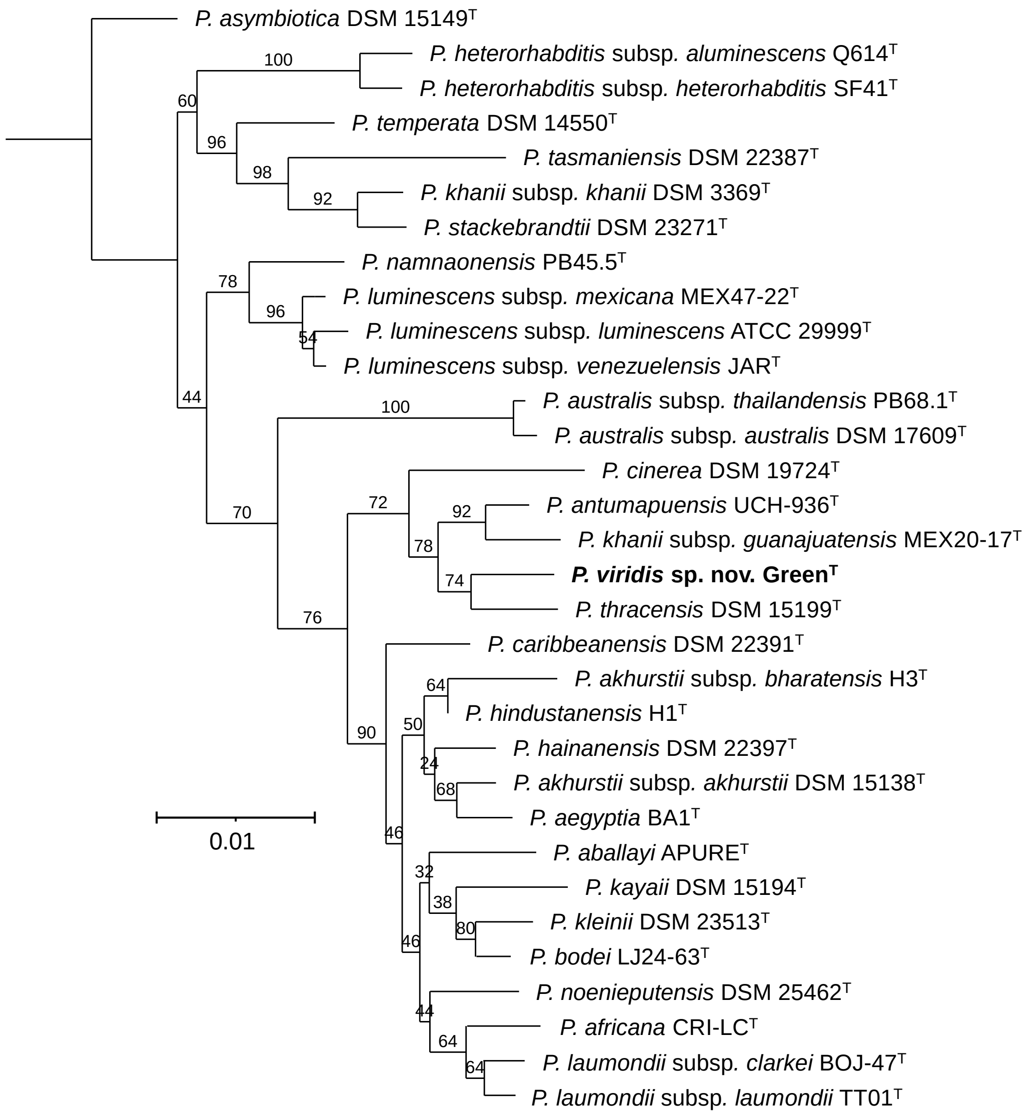

Figure S1. Maximum-likelihood phylogenetic tree reconstructed from 16S rRNA gene sequences of all *Photorhabdus* type strains with validly published names. The evolutionary history was inferred by using the Maximum Likelihood method based on the Kimura 2-parameter model. The percentage of trees in which the associated taxa clustered together is shown next to the branches. The tree is drawn to scale, with branch lengths measured in the number of substitutions per site. There were a total of 1221 positions in the final dataset. Evolutionary analyses were conducted in MEGA7 based on 100 replications. Accession numbers of gene sequences used are shown in Table S1.

	P. laumondii subsp. *laumondii* TT01^{T}	*P. laumondii* subsp. *clarkei* BOJ-47^{T}	*P. africana* CRI-LC^{T}	*P. noenieputensis* DSM 25462^{T}	*P. bodei* LJ24-63^{T}	*P. kleinii* DSM 23513^{T}	*P. kayaii* DSM 15194^{T}	*P. aballayi* APURE^{T}	*P. aegyptia* BA1^{T}	*P. akhurstii* subsp. *akhurstii* DSM 15138^{T}	*P. hainanensis* DSM 22397^{T}	*P. hindustanensis* H1^{T}	*P. akhurstii* subsp. *bharatensis* H3^{T}	*P. caribbeanensis* HG29^{T}	*P. thracensis* DSM 15199^{T}	***P. viridis* sp. nov. Green^{T}**	*P. khanii* subsp. *guanajuatensis* MEX20-17^{T}	*P. antumapuensis* UCH-936^{T}	*P. cinerea* DSM 19724^{T}	*P. australis* subsp. *australis* DSM 17609^{T}	*P. australis* subsp. *thailandensis* PB68.1^{T}	*P. luminescens subsp. venezuelensis* JAR^{T}	*P. luminescens* subsp. *luminescens* ATCC 29999^{T}	*P. luminescens* subsp. *mexicana* MEX47-22^{T}	*P. namnaonensis* PB45.5^{T}	*P. stackebrandtii* DSM 23271^{T}	*P. khanii* subsp. *khanii* DSM 3369^{T}	*P. tasmaniensis* DSM 22387^{T}	*P. temperata* DSM 14550^{T}	*P. heterorhabditis* subsp. *heterorhabditis* SF41^{T}	*P. heterorhabditis* subsp. *aluminescens* Q614^{T}	*P. asymbiotica* DSM 15149^{T}
P. asymbiotica DSM 15149^{T}	96.6	96.4	96.3	96.8	96.7	96.8	96.6	96.4	96.4	96.5	96.4	96.0	96.1	97.1	96.0	96.3	96.2	96.4	96.7	97.7	97.6	98.4	98.3	98.3	98.1	97.0	97.7	96.6	97.8	97.9	98.1	ID
P. heterorhabditis subsp. *aluminescens* Q614^{T}	96.4	96.3	96.2	96.3	96.2	96.0	95.9	96.1	96.4	96.1	96.4	96.0	96.1	96.8	96.9	97.3	96.8	97.0	97.0	97.3	97.3	97.7	97.7	97.5	97.6	97.6	98.2	97.4	98.3	99.2	ID	98.1
P. heterorhabditis subsp. *heterorhabditis* SF41^{T}	96.8	96.4	96.2	96.3	96.1	96.1	95.7	96.3	96.3	96.3	96.4	96.1	96.3	96.9	96.7	97.2	96.7	96.8	96.8	97.2	97.2	97.8	97.7	97.8	97.6	97.4	98.1	97.1	98.1	ID	99.2	97.9
P. temperata DSM 14550^{T}	96.2	96.2	96.4	96.5	96.7	96.4	96.1	96.6	96.4	96.1	96.8	96.0	96.1	96.8	97.2	97.3	97.3	97.2	97.5	97.0	97.0	98.1	98.1	98.0	97.7	98.0	98.3	97.5	ID	98.1	98.3	97.8
P. tasmaniensis DSM 22387^{T}	95.1	94.8	95.2	95.2	95.1	94.9	95.3	95.2	95.7	95.4	95.5	95.0	94.8	95.5	96.3	96.3	96.3	96.4	95.9	96.1	95.9	96.8	96.7	96.6	96.6	97.3	97.8	ID	97.5	97.1	97.4	96.6
P. khanii subsp. *khanii* DSM 3369^{T}	96.0	95.5	95.7	96.2	95.9	95.8	95.6	95.9	95.9	95.7	96.1	95.5	95.6	96.3	97.2	97.4	97.7	97.7	96.7	96.8	96.7	97.2	97.3	97.3	96.9	99.3	ID	97.8	98.3	98.1	98.2	97.7
P. stackebrandtii DSM 23271^{T}	96.0	95.9	96.0	96.5	96.2	96.1	95.7	96.0	95.9	95.7	96.4	95.7	95.9	96.3	97.2	97.2	98.0	98.1	96.8	96.3	96.2	96.8	97.0	97.0	96.8	ID	99.3	97.3	98.0	97.4	97.6	97.0
P. namnaonensis PB45.5^{T}	97.0	97.0	97.4	97.4	97.2	97.2	97.0	97.1	97.7	97.7	97.9	96.9	96.8	97.2	96.3	96.0	96.3	96.3	96.3	96.8	96.8	99.0	98.8	99.0	ID	96.8	96.9	96.6	97.7	97.6	97.6	98.1
P. luminescens subsp. *mexicana* MEX47-22^{T}	97.1	96.9	97.1	97.2	97.2	97.0	97.0	97.9	97.2	97.1	97.7	96.9	97.0	97.6	96.3	96.3	96.4	96.5	96.3	97.1	97.1	99.6	99.7	ID	99.0	97.0	97.3	96.6	98.0	97.8	97.5	98.3
P. luminescens subsp. *luminescens* ATCC 29999^{T}	96.8	96.7	97.0	97.0	97.1	96.8	96.9	97.8	97.2	96.9	97.7	96.8	96.8	97.5	96.4	96.3	96.4	96.5	96.4	97.0	97.0	99.7	ID	99.7	98.8	97.0	97.3	96.7	98.1	97.7	97.7	98.3
P. luminescens subsp. venezuelensis JAR^{T}	96.9	96.9	97.1	97.2	97.2	97.0	97.2	97.7	97.2	97.2	97.6	96.9	96.8	97.4	96.3	96.3	96.3	96.4	96.5	97.2	97.2	ID	99.7	99.6	99.0	96.8	97.2	96.8	98.1	97.8	97.7	98.4
P. australis subsp. *thailandensis* PB68.1^{T}	97.2	97.1	97.0	97.2	96.8	97.1	96.8	96.8	97.2	97.0	97.3	96.7	97.0	97.7	96.5	96.8	96.8	97.0	97.4	99.8	ID	97.2	97.0	97.1	96.8	96.2	96.7	95.9	97.0	97.2	97.3	97.6
P. australis subsp. *australis* DSM 17609^{T}	97.2	97.0	96.9	97.2	96.8	97.2	96.8	96.7	97.1	96.9	97.2	96.6	96.9	97.7	96.4	96.8	96.8	97.1	97.5	ID	99.8	97.2	97.0	97.1	96.8	96.3	96.8	96.1	97.0	97.2	97.3	97.7
P. cinerea DSM 19724^{T}	97.5	97.7	97.5	97.9	97.9	97.7	97.7	97.9	97.8	97.5	97.7	97.1	97.5	97.7	97.9	98.1	98.5	98.7	ID	97.5	97.4	96.5	96.4	96.3	96.3	96.8	96.7	95.9	97.5	96.8	97.0	96.7
P. antumapuensis UCH-936^{T}	97.8	97.7	97.5	98.2	98.0	97.9	97.6	97.9	97.8	97.4	98.0	97.3	97.6	97.9	98.6	98.6	99.7	ID	98.7	97.1	97.0	96.4	96.5	96.5	96.3	98.1	97.7	96.4	97.2	96.8	97.0	96.4
P. khanii subsp. *guanajuatensis* MEX20-17^{T}	97.7	97.6	97.4	98.0	97.9	97.8	97.3	97.8	97.7	97.3	97.9	97.2	97.5	97.8	98.6	98.5	ID	99.7	98.5	96.8	96.8	96.3	96.4	96.4	96.3	98.0	97.7	96.3	97.3	96.7	96.8	96.2
***P. viridis* sp. nov. Green^{T}**	97.7	97.2	96.9	97.9	97.5	97.2	96.9	97.7	97.8	97.5	97.7	97.3	97.3	97.8	98.8	ID	98.5	98.6	98.1	96.8	96.8	96.3	96.3	96.3	96.0	97.2	97.4	96.3	97.3	97.2	97.3	96.3
P. thracensis DSM 15199^{T}	97.4	96.9	96.9	97.5	97.6	97.3	97.1	97.7	98.1	97.7	97.9	97.3	97.1	97.7	ID	98.8	98.6	98.6	97.9	96.4	96.5	96.3	96.4	96.3	96.3	97.2	97.2	96.3	97.2	96.7	96.9	96.0
P. caribbeanensis HG29^{T}	98.8	98.5	98.5	98.5	98.6	98.3	97.7	98.5	98.8	98.3	98.8	98.1	98.3	ID	97.7	97.8	97.8	97.9	97.7	97.7	97.7	97.4	97.5	97.6	97.2	96.3	96.3	95.5	96.8	96.9	96.8	97.1
P. akhurstii subsp. *bharatensis* H3^{T}	98.3	98.3	97.8	98.3	98.4	98.1	97.5	98.1	98.3	98.4	98.6	98.4	ID	98.3	97.1	97.3	97.5	97.6	97.5	96.9	97.0	96.8	96.8	97.0	96.8	95.9	95.6	94.8	96.1	96.3	96.1	96.1
P. hindustanensis H1^{T}	98.3	98.1	97.8	98.1	98.1	98.0	97.7	98.1	98.4	98.7	98.5	ID	98.4	98.1	97.3	97.3	97.2	97.3	97.1	96.6	96.7	96.9	96.8	96.9	96.9	95.7	95.5	95.0	96.0	96.1	96.0	96.0
P. hainanensis DSM 22397^{T}	98.3	98.1	98.5	98.6	98.7	98.5	98.1	98.6	99.3	98.7	ID	98.5	98.6	98.8	97.9	97.7	97.9	98.0	97.7	97.2	97.3	97.6	97.7	97.7	97.9	96.4	96.1	95.5	96.8	96.4	96.4	96.4
P. akhurstii subsp. *akhurstii* DSM 15138^{T}	98.4	98.1	98.1	98.3	98.5	98.1	97.8	98.1	99.2	ID	98.7	98.7	98.4	98.3	97.7	97.5	97.3	97.4	97.5	96.9	97.0	97.2	96.9	97.1	97.7	95.7	95.7	95.4	96.1	96.3	96.1	96.5
P. aegyptia BA1^{T}	98.6	98.3	98.5	98.6	98.9	98.5	98.2	98.5	ID	99.2	99.3	98.4	98.3	98.8	98.1	97.8	97.7	97.8	97.8	97.1	97.2	97.2	97.2	97.2	97.7	95.9	95.9	95.7	96.4	96.3	96.4	96.4
P. aballayi APURE^{T}	98.6	98.5	98.4	98.6	98.6	98.2	98.6	ID	98.5	98.1	98.6	98.1	98.1	98.5	97.7	97.7	97.8	97.9	97.9	96.7	96.8	97.7	97.8	97.9	97.1	96.0	95.9	95.2	96.6	96.3	96.1	96.4
P. kayaii DSM 15194^{T}	98.3	98.5	98.3	98.5	98.6	98.7	ID	98.6	98.2	97.8	98.1	97.7	97.5	97.7	97.1	96.9	97.3	97.6	97.7	96.8	96.8	97.2	96.9	97.0	97.0	95.7	95.6	95.3	96.1	95.7	95.9	96.6
P. kleinii DSM 23513^{T}	98.5	98.6	98.3	98.7	99.4	ID	98.7	98.2	98.5	98.1	98.5	98.0	98.1	98.3	97.3	97.2	97.8	97.9	97.7	97.2	97.1	97.0	96.8	97.0	97.2	96.1	95.8	94.9	96.4	96.1	96.0	96.8
P. bodei LJ24-63^{T}	98.4	98.6	98.6	98.6	ID	99.4	98.6	98.6	98.9	98.5	98.7	98.1	98.4	98.6	97.6	97.5	97.9	98.0	97.9	96.8	96.8	97.2	97.1	97.2	97.2	96.2	95.9	95.1	96.7	96.1	96.2	96.7
P. noenieputensis DSM 25462^{T}	99.0	99.0	98.6	ID	98.6	98.7	98.5	98.6	98.6	98.3	98.6	98.1	98.3	98.5	97.5	97.9	98.0	98.2	97.9	97.2	97.2	97.2	97.0	97.2	97.4	96.5	96.2	95.2	96.5	96.3	96.3	96.8
P. africana CRI-LC^{T}	99.0	99.1	ID	98.6	98.6	98.3	98.3	98.4	98.5	98.1	98.5	97.8	97.8	98.5	96.9	96.9	97.4	97.5	97.5	96.9	97.0	97.1	97.0	97.1	97.4	96.0	95.7	95.2	96.4	96.2	96.2	96.3
P. laumondii subsp. *clarkei* BOJ-47^{T}	99.5	ID	99.1	99.0	98.6	98.6	98.5	98.5	98.3	98.1	98.1	98.1	98.3	98.5	96.9	97.2	97.6	97.7	97.7	97.0	97.1	96.9	96.7	96.9	97.0	95.9	95.5	94.8	96.2	96.4	96.3	96.4
P. laumondii subsp. *laumondii* TT01^{T}	ID	99.5	99.0	99.0	98.4	98.5	98.3	98.6	98.6	98.4	98.3	98.3	98.3	98.8	97.4	97.7	97.7	97.8	97.5	97.2	97.2	96.9	96.8	97.1	97.0	96.0	96.0	95.1	96.2	96.8	96.4	96.6

Figure S2. Pairwise nucleotide similarities (%) of 16S rRNA gene sequences of all *Photorhabdus* type strains with validly published names. A total of 1221 nucleotide positions were analyzed. Accession numbers of gene sequences used are shown in Table S1.

	P. asymbiotica DSM 15149^{T}	*P. australis* subsp. *thailandensis* PB68.1^{T}	*P. australis* subsp. *australis* DSM 17609^{T}	*P. cinerea* DSM 19724^{T}	*P. heterorhabditis* subsp. *heterorhabditis* SF41^{T}	*P. heterorhabditis* subsp. *aluminescens* Q614^{T}	*P. stackebrandtii* DSM 23271^{T}	*P. khanii* subsp. *khanii* DSM 3369^{T}	*P. khanii* subsp. *guanajuatensis* MEX20-17^{T}	*P. tasmaniensis* DSM 22387^{T}	***P. viridis* sp. nov. Green^{T}**	*P. thracensis* DSM 15199^{T}	*P. temperata* DSM 14550^{T}	*P. antumapuensis* UCH-936^{T}	*P. africana* CRI-LC^{T}	*P. laumondii* subsp. *laumondii* TT01^{T}	*P. laumondii* subsp. *clarkei* BOJ-47^{T}	*P. kayaii* DSM 15194^{T}	*P. kleinii* DSM 23513^{T}	*P. bodei* LJ24-63^{T}	*P. aballayi* sp. nov. APURE^{T}	*P. luminescens* subsp. *luminescens* ATCC 29999^{T}	*P. luminescens* subsp. *mexicana* MEX47-22^{T}	*P. luminescens* subsp. *venezuelensis* JAR^{T}	*P. noenieputensis* DSM 25462^{T}	*P. caribbeanensis* HG29^{T}	*P. aegyptia* BA1^{T}	*P. akhurstii* subsp. *bharatensis* H3^{T}	*P. akhurstii* subsp. *akhurstii* DSM 15138^{T}	*P. hainanensis* DSM 22397^{T}	*P. hindustanensis* H1^{T}	*P. namnaonensis* PB45.5^{T}
P. asymbiotica DSM 15149^{T}	ID	92.4	92.5	90.7	90.6	90.5	85.9	85.7	85.8	85.5	85.5	85.9	85.6	85.9	85.7	85.6	85.5	85.6	85.6	85.7	85.5	85.6	85.6	85.6	85.4	85.3	85.5	85.3	85.4	85.2	85.3	85.1
P. australis subsp. *thailandensis* PB68.1^{T}	92.4	ID	97.3	91.2	91.1	91.2	85.5	85.4	85.3	85.5	85.6	85.7	85.5	85.5	85.6	85.7	85.6	85.6	85.4	85.7	85.1	85.2	85.2	85.1	85.6	85.4	85.7	85.6	85.7	85.7	85.6	85.6
P. australis subsp. *australis* DSM 17609^{T}	92.5	97.4	ID	91.2	91.3	91.5	85.4	85.4	85.4	85.5	85.3	85.6	85.6	85.5	85.6	85.8	85.6	85.5	85.5	85.7	85.1	85.2	85.2	85.2	85.6	85.3	85.5	85.5	85.6	85.6	85.5	85.3
P. cinerea DSM 19724^{T}	90.8	91.2	91.2	ID	94.7	94.8	86.3	86.4	86.3	86.3	86.3	86.5	87.0	86.2	85.9	86.1	85.9	86.1	85.9	86.2	85.4	85.5	85.5	85.4	85.6	85.5	85.7	85.7	85.7	85.7	85.7	85.5
P. heterorhabditis subsp. *heterorhabditis* SF41^{T}	90.6	91.2	91.3	94.7	ID	97.2	86.1	86.0	86.1	86.1	86.5	86.3	86.4	86.1	86.1	86.1	86.0	86.0	85.7	86.2	85.4	85.5	85.5	85.5	86.0	85.5	85.7	85.9	85.6	85.7	85.5	85.5
P. heterorhabditis subsp. *aluminescens* Q614^{T}	90.6	91.3	91.5	94.8	97.3	ID	86.2	86.1	86.1	86.5	86.4	86.5	86.4	86.1	86.0	86.2	85.9	86.1	85.6	86.2	85.3	85.5	85.4	85.6	86.0	85.5	85.8	85.8	85.8	86.0	85.6	85.7
P. stackebrandtii DSM 23271^{T}	85.7	85.5	85.4	86.3	86.1	86.1	ID	95.9	94.4	92.2	92.6	93.3	93.2	87.4	86.4	86.3	86.2	86.5	86.7	86.5	86.0	86.1	86.1	86.4	86.1	85.9	86.0	85.9	85.8	85.8	85.8	85.9
P. khanii subsp. *khanii* DSM 3369^{T}	85.7	85.3	85.3	86.2	85.9	86.1	95.9	ID	96.6	92.2	92.5	93.2	93.1	87.6	86.4	86.3	86.5	86.6	86.9	86.6	86.1	86.2	86.2	86.2	86.1	86.0	86.2	86.1	86.0	86.0	86.0	85.9
P. khanii subsp. *guanajuatensis* MEX20-17^{T}	85.7	85.3	85.4	86.2	86.1	86.0	94.4	96.7	ID	92.3	92.4	93.1	93.2	87.6	86.2	86.3	86.3	86.5	86.8	86.6	86.1	86.1	86.3	86.1	86.2	85.9	86.2	86.1	86.0	85.9	86.1	85.9
P. tasmaniensis DSM 22387^{T}	85.4	85.4	85.4	86.2	86.1	86.4	92.1	92.2	92.4	ID	92.4	93.1	93.1	86.8	86.1	86.1	86.2	86.3	86.2	86.4	85.6	85.8	85.6	85.6	85.9	85.7	85.9	85.9	85.7	85.8	85.9	85.8
***P. viridis* sp. nov. Green^{T}**	85.5	85.4	85.4	86.3	86.5	86.3	92.5	92.6	92.7	92.5	ID	94.5	94.7	86.8	86.4	86.3	86.3	86.7	86.3	86.5	85.7	86.1	85.8	85.9	86.2	85.9	86.2	86.1	86.0	86.2	86.1	85.9
P. thracensis DSM 15199^{T}	85.7	85.8	85.6	86.5	86.4	86.4	93.3	93.2	93.3	93.1	94.4	ID	95.9	87.0	86.7	86.4	86.6	87.2	86.8	86.9	85.9	86.0	85.9	85.9	86.4	85.9	86.2	86.2	86.1	86.2	86.1	86.1
P. temperata DSM 14550^{T}	85.5	85.6	85.5	86.9	86.4	86.3	93.1	93.2	93.3	93.2	94.6	95.9	ID	86.9	86.3	86.2	86.3	86.5	86.3	86.5	85.8	86.0	85.8	85.9	86.0	85.9	86.1	86.0	86.1	86.0	86.1	85.9
P. antumapuensis UCH-936^{T}	85.9	85.4	85.6	86.2	86.1	85.9	87.3	87.7	87.8	86.8	86.8	87.0	86.9	ID	92.4	92.3	92.3	92.6	93.1	92.6	91.9	92.0	91.9	92.2	91.3	91.1	91.2	91.0	90.9	90.9	91.0	90.8
P. africana CRI-LC^{T}	85.7	85.7	85.8	86.1	86.3	86.2	86.5	86.5	86.4	86.2	86.4	86.6	86.4	92.4	ID	95.7	95.4	94.7	93.8	94.4	91.4	91.5	91.3	91.5	92.6	92.1	92.0	91.9	92.0	91.8	91.7	91.5
P. laumondii subsp. *laumondii* TT01^{T}	85.7	85.8	85.8	86.1	86.2	86.2	86.3	86.3	86.3	86.1	86.4	86.5	86.2	92.2	95.8	ID	97.6	94.7	93.7	94.5	91.3	91.4	91.3	91.5	92.8	92.3	92.3	91.9	92.0	91.8	91.8	91.5
P. laumondii subsp. *clarkei* BOJ-47^{T}	85.6	85.8	85.6	85.9	86.0	85.9	86.3	86.5	86.5	86.3	86.4	86.6	86.3	92.2	95.5	97.5	ID	94.7	93.8	94.9	91.3	91.4	91.4	91.5	92.6	92.3	92.5	92.5	92.5	92.2	92.4	91.9
P. kayaii DSM 15194^{T}	85.7	85.8	85.7	86.2	86.2	86.1	86.5	86.6	86.6	86.5	86.7	87.2	86.6	92.5	94.7	94.7	94.8	ID	95.6	96.3	91.4	91.5	91.5	91.5	92.4	92.2	92.2	92.2	92.1	92.0	91.9	91.8
P. kleinii DSM 23513^{T}	85.6	85.4	85.6	85.8	85.7	85.7	86.7	86.9	86.8	86.2	86.4	86.7	86.5	93.0	93.8	93.7	93.9	95.6	ID	96.5	91.9	92.3	92.2	92.3	92.2	92.0	92.1	91.9	91.9	91.8	91.8	91.5
P. bodei LJ24-63^{T}	85.7	85.8	85.8	86.4	86.3	86.3	86.5	86.6	86.5	86.4	86.5	86.9	86.8	92.5	94.4	94.5	94.8	96.3	96.5	ID	91.5	91.6	91.6	91.5	92.4	92.2	92.3	92.5	92.5	92.4	92.5	92.2
P. aballayi sp. nov. APURE^{T}	85.4	85.1	85.2	85.5	85.3	85.3	86.0	86.1	86.2	85.6	85.8	85.9	85.8	91.8	91.3	91.3	91.3	91.4	92.0	91.4	ID	95.5	95.4	95.9	91.9	91.9	91.8	91.6	91.6	91.4	91.3	91.2
P. luminescens subsp. *luminescens* ATCC 29999^{T}	85.4	85.2	85.3	85.5	85.5	85.5	86.0	86.2	86.3	85.9	86.0	86.0	86.0	92.0	91.4	91.3	91.3	91.5	92.2	91.5	95.5	ID	96.8	97.0	92.1	92.1	92.1	91.8	91.8	91.7	91.6	91.5
P. luminescens subsp. *mexicana* MEX47-22^{T}	85.4	85.1	85.2	85.5	85.4	85.3	85.9	86.3	86.2	85.7	85.8	85.9	85.8	91.8	91.4	91.3	91.4	91.4	92.1	91.4	95.4	96.9	ID	97.3	92.2	92.0	91.9	91.9	91.7	91.6	91.6	91.4
P. luminescens subsp. *venezuelensis* JAR^{T}	85.5	85.2	85.3	85.5	85.5	85.4	86.3	86.2	86.2	85.7	85.8	86.0	85.8	92.1	91.4	91.4	91.5	91.5	92.2	91.5	95.9	97.1	97.3	ID	92.1	91.9	91.9	91.7	91.7	91.5	91.6	91.4
P. noenieputensis DSM 25462^{T}	85.3	85.4	85.5	85.6	86.0	85.9	86.0	86.0	86.2	85.8	86.2	86.2	86.0	91.4	92.6	92.8	92.6	92.4	92.2	92.4	91.9	92.2	92.2	92.2	ID	94.7	94.2	93.5	93.5	93.2	93.2	92.8
P. caribbeanensis HG29^{T}	85.2	85.3	85.2	85.6	85.5	85.4	85.8	85.9	86.0	85.5	85.8	85.9	85.8	91.0	92.2	92.3	92.3	92.1	91.9	92.2	91.9	92.0	92.0	91.9	94.7	ID	94.9	93.7	93.7	93.4	93.3	92.9
P. aegyptia BA1^{T}	85.4	85.6	85.4	85.8	85.6	85.6	85.9	86.2	86.1	85.9	86.1	86.1	86.2	91.1	92.0	92.2	92.4	92.2	92.0	92.3	91.7	92.0	92.0	91.9	94.1	94.8	ID	96.3	96.6	95.5	95.8	94.5
P. akhurstii subsp. *bharatensis* H3^{T}	85.2	85.4	85.4	85.7	85.6	85.6	85.8	86.1	86.1	85.8	86.0	86.0	85.9	90.9	91.8	91.9	92.5	92.0	91.8	92.3	91.5	91.8	91.8	91.7	93.5	93.7	96.3	ID	97.7	96.6	96.6	95.1
P. akhurstii subsp. *akhurstii* DSM 15138^{T}	85.2	85.4	85.4	85.7	85.6	85.6	85.8	86.0	86.0	85.7	85.9	86.1	85.9	90.9	91.8	91.9	92.4	92.0	91.9	92.4	91.5	91.7	91.7	91.5	93.4	93.6	96.6	97.7	ID	96.5	96.9	95.3
P. hainanensis DSM 22397^{T}	85.1	85.5	85.6	85.7	85.6	85.7	85.8	86.0	86.0	85.9	86.1	86.0	86.1	90.8	91.8	91.7	92.2	91.9	91.8	92.2	91.4	91.7	91.6	91.6	93.2	93.4	95.4	96.7	96.7	ID	96.4	95.4
P. hindustanensis H1^{T}	85.0	85.4	85.3	85.7	85.4	85.4	85.8	86.0	86.0	85.9	85.9	86.0	86.0	91.0	91.7	91.9	92.4	91.9	91.7	92.3	91.4	91.6	91.7	91.5	93.1	93.2	95.8	96.6	96.9	96.4	ID	95.8
P. namnaonensis PB45.5^{T}	85.1	85.3	85.4	85.5	85.4	85.6	85.8	85.9	85.9	85.9	85.9	86.0	86.0	90.8	91.5	91.4	91.9	91.6	91.5	92.0	91.2	91.4	91.4	91.4	92.6	92.8	94.5	95.1	95.3	95.4	95.8	ID

Figure S3. Pairwise comparisons of average nucleotide identity (ANI) values (%) of all *Photorhabdus* type strains with validly published names. Accession numbers of gene sequences used are shown in Table S1.

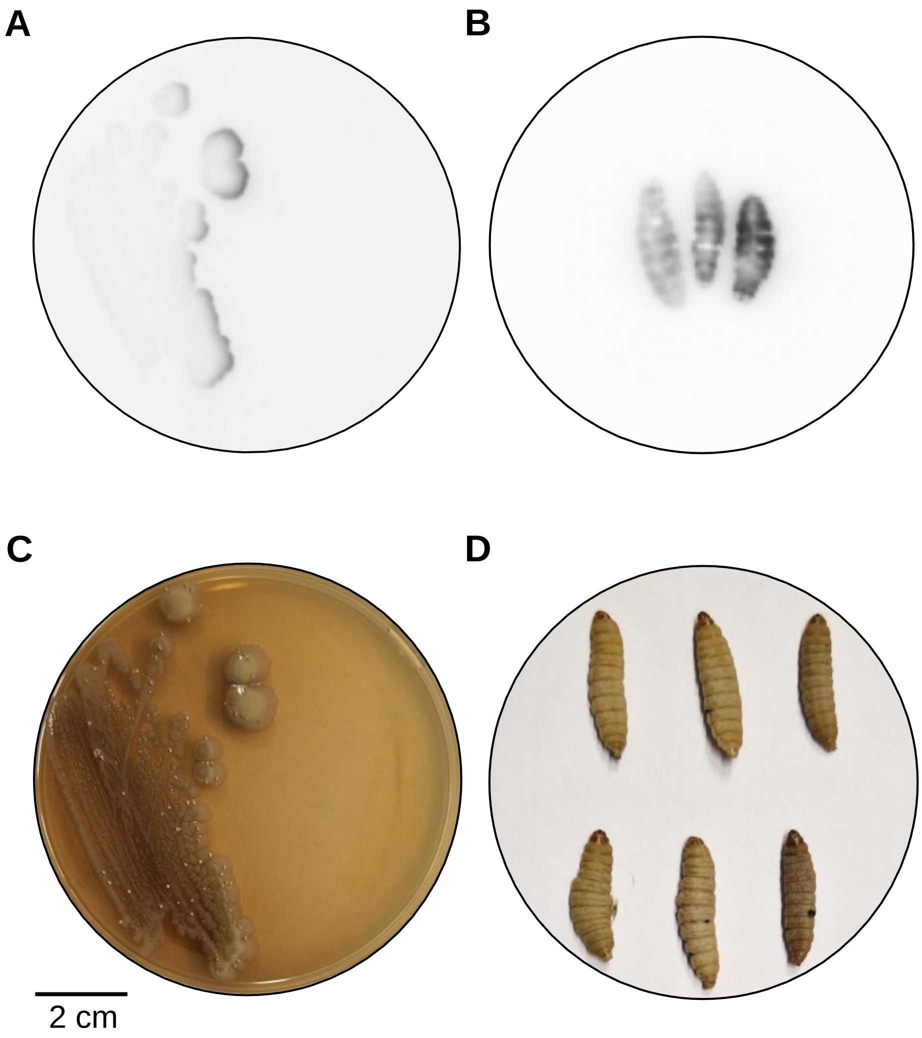

Figure S4. Bioluminescence emitted by A) *Photorhabdus viridis* sp. nov. Green^{T} cultured on LB medium or B) *Galleria mellonella* larvae infested by *Heterorhabditis zealandica* MJ2C nematodes 4 days after infestation. Darker colours indicate higher bioluminescence levels. Photographs were made using an Amersham Imager 600 (Cytiva, US). C) Colony morphology of *P. viridis* sp. nov. Green^{T} cultured on LB medium for 3 weeks at 24°C. D) *G. mellonella* larvae infested by *H. zealandica* MJ2C nematodes 4 days after nematode inoculation.

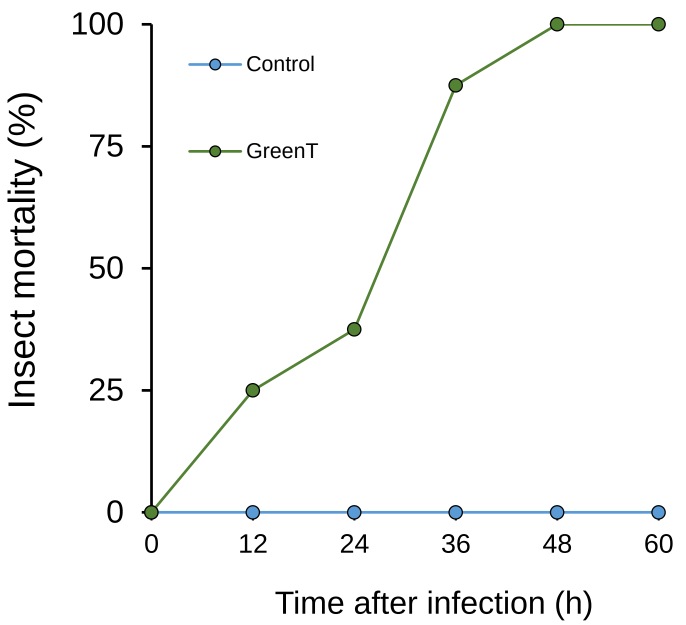

Figure S4. Entomopathogenicity of *P. viridis* sp. nov. Green^{T}. Insect mortality (%) caused by injecting the different bacterial strains at densities of OD_{600}=0.01.

-SUPPLEMENTARY TABLES-

Table S1. National Center for Biotechnology Information (NCBI) accession numbers of the sequences used in this study. Sequences generated in this study are shown in bold.

Strain	16S	Genome
P. aballayi APURE^T	OP735347	JAPFCD01
P. africana CRI-LC^T	OR835571	JAXBVE01
P. aegyptia BA1^T	MT355495	JFGV01
P. akhurstii subsp. *akhurstii* DSM 15138^T	MK039086	RCWE01
P. akhurstii subsp. *bharatensis* H3^T	KJ995730	PUWU01
P. antumapuensis UCH-936^T	MZ676562	JAHZMK01
P. australis subsp. *thailandensis* PB68.1^T	MT355494	LOMY01
P. australis subsp. *australis* DSM 17609^T	AY280572	JONO01
P. asymbiotica ATCC 43949^T	Z76752	RBLJ01
P. bodei LJ24-63^T	MK039080	NSCM01
P. caribbeanensis DSM 22391^T	MK039083	RCWB01
P. cinerea DSM 19724^T	MK039069	PUJW01
P. hainanensis DSM 22397^T	MK039085	RCWD01
P. heterorhabditis subsp. *aluminescens* Q614^T	AY216500	JABBCS01
P. heterorhabditis subsp. *heterorhabditis* SF41^T	MK039068	RCWA01
P. hindustanensis H1^T	JX221722	PUWT01
P. kayaii DSM 15194^T	MK039081	JAJAFZ01
P. khanii subsp. *khanii* DSM 3369^T	MK039076	AYSJ01
P. khanii subsp. *guanajuatensis* MEX20-17^T	MK053912	PUJY01
P. kleinii DSM 23513^T	MK039079	JAJAFY01
P. laumondii subsp. *clarkei* BOJ-47^T	MK039078	NSCI01
P. laumondii subsp. *laumondii* TT01^T	MK039077	WSFH01
P. luminescens subsp. *luminescens* ATCC 29999^T	MK039082	FMWJ01
P. luminescens subsp. *mexicana* MEX47-22^T	MK053913	PUJX01
P. luminescens subsp. *venezuelensis* JAR^T	OP727818	JAPFFZ01
P. namnaonensis PB45.5^T	MK039087	LOIC01
P. noenieputensis DSM 25462^T	MK039084	RCWC01
P. stackebrandtii DSM 23271^T	MK039075	PUJV01
P. tasmaniensis DSM 22387^T	MK039072	PUJU01
P. temperata DSM 14550^T	MK039073	JAJAFX01
P. thracensis DSM 15199^T	MK039074	CP011104
Photorhabdus viridis **sp. nov. Green**^{T}	**PP911496**	**JBEJZY01**

Table S2. Features of the *Photorhabdus* genomes used in this study.

Scientific name	**Base pairs**	**Percent G+C**	**No. proteins**
P. aballayi APURE^T	5656459	42.42	4932
P. africana CRI-LC^T	5200517	42.79	4560
P. aegyptia BA1^T	5004588	42.46	4259
P. akhurstii subsp. *akhurstii* DSM 15138^T	5552363	42.79	4799
P. akhurstii subsp. *bharatensis* H3^T	5514710	42.71	5043
P. antumapuensis UCH-936^T	5046591	42.52	4398
P. asymbiotica ATCC 43949^T	5103671	42.19	4397
P. australis subsp. *thailandensis* PB68.1^T	4800313	42.10	4301
P. australis subsp. *australis* DSM 17609^T	4912527	42.06	4436
P. bodei LJ24-63^T	5059726	42.71	4559
P. caribbeanensis DSM 22391^T	5362644	42.43	4651
P. cinerea DSM 19724^T	4899918	42.26	4613
P. hainanensis DSM 22397^T	5492441	42.83	4820
P. heterorhabditis subsp. *aluminescens* Q614^T	4981871	42.61	4660
P. heterorhabditis subsp. *heterorhabditis* SF41^T	5052136	42.24	4625
P. hindustanensis H1^T	5663704	42.86	5025
P. kayaii DSM 15194^T	5121090	42.54	4619
P. khanii subsp. *guanajuatensis* MEX20-17^T	5512522	43.52	4925
P. khanii subsp. *khanii* DSM 3369^T	5232343	43.54	4554
P. kleinii DSM 23513^T	5162879	42.41	4606
P. laumondii subsp. *clarkei* BOJ-47^T	5109397	42.52	4474
P. laumondii subsp. *laumondii* TT01^T	5376124	42.58	4721
P. luminescens subsp. *luminescens* ATCC 29999^T	5293870	42.60	4548
P. luminescens subsp. *mexicana* MEX47-22^T	5827786	42.53	5075
P. luminescens subsp. *venezuelensis* JAR^T	5429594	42.46	4585
P. namnaonensis PB45.5^T	5420031	42.70	4678
P. noenieputensis DSM 25462^T	5370579	42.56	4798
P. stackebrandtii DSM 23271^T	4846662	43.12	4427
P. tasmaniensis DSM 22387^T	5163014	43.56	4771
P. temperata DSM 14550^T	5382617	43.44	4872
P. thracensis DSM 15199^T	5147098	44.11	4542
***Photorhabdus viridis* sp. nov. Green^T**	5122085	43.64	5115

Table S3. Features of the genomes of *Photorhabdus viridis* sp. nov. Green^T.

	**Green^T**
Scaffold L50	23
Scaffold N50	78106
Scaffold L90	79
Scaffold N90	9166
Scaffold len_max	185201
Scaffold len_min	112
Scaffold len_mean	5597
Scaffold len_median	243
Scaffold len_std	20471
Scaffold num_A	1450769
Scaffold num_T	1436265
Scaffold num_C	1127294
Scaffold num_G	1107757
Scaffold num_N	0
Scaffold num_bp	5122085
Scaffold num_bp_not_N	5122085
Scaffold num_seq	915
Scaffold GC content overall	43.64

Table S4. Antibiotic-resistance conferring genes of *Photorhabdus viridis* sp. nov. Green^{T}, and their more closely related strains: *P. tasmaniensis* DSM 22387^{T}, *P. thracensis* DSM 15199^{T}, and *P. temperata* DSM 14550^{T}. (+): present; (-): absent or non-functional.

Gene	Resistance mechanism	AMR Gene Family	Drug Class	**Green^{T}**	DSM 22387^{T}	DSM 15199^{T}	DSM 14550^{T}
AdeF	Antibiotic efflux	Resistance-nodulation-cell division (RND) antibiotic efflux pump	Fluoroquinolones, tetracyclines	**+**	+	+	+
CRP			Macrolides, fluoroquinolones, penam	**+**	+	+	+
rsmA			Fluoroquinolones, diaminopyrimidines, phenicols	**+**	+	+	+
KpnH		Major facilitator superfamily (MFS) antibiotic efflux pump	Macrolides, fluoroquinolones, aminoglycosides, carbapenems, cephalosporins, penams, peptides, penems	**+**	+	+	+
KpnF			Macrolides, aminoglycosides, cephalosporins, tetracycline, peptides, rifamycin.	**+**	+	+	+
qacJ		Small multidrug resistance (SMR) antibiotic efflux pump	Disinfecting agents and antiseptics	**+**	+	+	+
EF-Tu	Antibiotic target alteration	Elfamycin resistant EF-Tu	Elfamycins	**-**	+	+	-
PBP3		Penicillin-binding protein mutations conferring resistance to beta-lactam antibiotics	Cephalosporins, cephamycins, penams	**+**	+	+	+
ArnT		Phosphoethanolamine transferase	Polymyxin peptides	**+**	+	+	+

Table S5. Predicted specialized metabolites produced by *Photorhabdus viridis* sp. nov. Green^T, and their more closely related strains: *P. tasmaniensis* DSM 22387^T, *P. thracensis* DSM 15199^T, and *P. temperata* DSM 14550^T*.* (+): Produced; (-): Not produced.

Specialized metabolite	**Green^T**	DSM 22387^T	DSM 15199^T	DSM 14550^T
Ambactin	+	-	-	-
Andrimid	+	-	-	+
Arthrofactin A	-	-	-	-
Bicornutin A1-A2	-	+	-	+
Bovienimide A	+	-	-	-
Carotenoid	-	-	-	-
Darobactin A	-	-	+	-
Fosfomycin	-	-	-	-
Gamexpeptide C	-	+	-	-
Holomycin	+	-	-	+
Jessenipeptin	-	+	-	-
Kolossin	+	+	-	+
Indigoidine	-	-	-	-
Luminmide	-	-	-	-
Luminmycin A	-	-	-	-
Minimycin	-	-	+	+
Odilorhabdins	-	-	-	-
Photobactin	+	+	+	+
Putrebactin	-	-	-	-
Piscibactin	-	-	-	-
Pyrrolizixenamide A	+	+	+	+
Rhizomide A-C	+	-	-	-
Ririwpeptide A-C	+	+	-	+
Stechlisins	+	-	-	-
TaxIllaid A	-	-	-	-
Tolaasin A	-	-	-	-
Turnerbactin	-	-	-	-
Xenematide	-	-	-	-
Xenocoumacin I-II	-	-	-	-
Xenortide A-D	-	-	+	-
Xenotetrapeptide	-	-	-	-
